# A meta-ethnographic review of people's experience of seeking asylum in the UK and its impact on psychological and social wellbeing

**DOI:** 10.1192/bjo.2021.670

**Published:** 2021-06-18

**Authors:** Christine Fullerton

**Affiliations:** Queen Mary University of London, Barts and the London School of Medicine and Dentistry

## Abstract

**Aims:**

Ethnographic accounts of the everyday, lived experience of seeking asylum have been incredibly useful for shedding light on how the asylum process and UK policy influences health and wellbeing. However, there lacks an analysis which pulls together these voices and establishes common themes. This review aims to address this gap by synthesising published literature related to people's experience of seeking asylum in the UK and its impact on their psychological and social wellbeing.

**Method:**

A systematic literature search was conducted in SCOPUS, PubMed and PsychINFO. Ten qualitative studies, capturing the accounts of over 190 people, were included in the review. The steps of meta-ethnography were used to synthesise the experiences of seeking asylum. Overarching themes which linked the studies were conceptualised and a framework of ‘constructs’ used to organise verbatim narratives and researcher interpretations from each study by theme and sub-theme. Finally, the constructs from each theme were translated to produce an overarching line of argument to the research.

**Result:**

Five key themes illustrating the experience of seeking asylum in the UK were identified. These were: a need for safety; distress; resilience and coping; sources of support; and looking to the future. The line of argument indicated that people seeking asylum in the UK experience a need for safety, high levels of psychological distress and social isolation, yet throughout exhibit extreme resilience. Analysis highlighted the need for increased governmental support and legal empowerment during the asylum process.

**Conclusion:**

This synthesis illustrates the widespread impact, both direct and indirect, of a culture of deterrence and disbelief within the Home Office on the psychological and social wellbeing of people desperately seeking refuge and compassion. To achieve equitable and optimum health for those seeking asylum in the UK, we must urgently move away from the hostile environment which has been created. As we develop a more holistic and expanded notion of health, the concept of wellbeing provides a person-centred framework for understanding how the social context can result in certain outcomes. The global public health response to the health-needs of people seeking asylum, and the wider migrant community, must be informed by lived experiences if they are to create interventions which have benefit.

